# 3D visualization of XFEL beam focusing properties using LiF crystal X-ray detector

**DOI:** 10.1038/srep17713

**Published:** 2015-12-04

**Authors:** Tatiana Pikuz, Anatoly Faenov, Takeshi Matsuoka, Satoshi Matsuyama, Kazuto Yamauchi, Norimasa Ozaki, Bruno Albertazzi, Yuichi Inubushi, Makina Yabashi, Kensuke Tono, Yuya Sato, Hirokatsu Yumoto, Haruhiko Ohashi, Sergei Pikuz, Alexei N. Grum-Grzhimailo, Masaharu Nishikino, Tetsuya Kawachi, Tetsuya Ishikawa, Ryosuke Kodama

**Affiliations:** 1 PPC and GSE Osaka University, Suita, Osaka 565-0871, Japan; 2 Joint Institute for High Temperatures, Russian Academy of Sciences, Moscow 125412, Russia; 3 Institute for Academic Initiatives, Osaka University, Suita, Osaka 565-0871, Japan; 4 Department of Precision Science and Technology, Graduate School of Engineering, Osaka University, Suita, Osaka 565-0871, Japan; 5 Center for Ultra-Precision Science and Technology, Graduate School of Engineering, Osaka University, Suita, Osaka 565-1871, Japan; 6 Japan Synchrotron Radiation Research Institute (JASRI), Sayo, Hyogo 679-5198, Japan; 7 RIKEN SPring-8 Center, Sayo, Hyogo 679-5148, Japan; 8 Division of Electrical, Electronic and Information Engineering, Graduate School of Engineering, Osaka University, Suita, Osaka 565-0871, Japan; 9 Skobeltsyn Institute of Nuclear Physics, Lomonosov Moscow State University, Moscow 119991, Russia; 10 Quantum Beam Science Center, Japan Atomic Energy Agency, Kizugawa, Kyoto 619-0215, Japan

## Abstract

Here, we report, that by means of direct irradiation of lithium fluoride a (LiF) crystal, *in situ* 3D visualization of the SACLA XFEL focused beam profile along the propagation direction is realized, including propagation inside photoluminescence solid matter. High sensitivity and large dynamic range of the LiF crystal detector allowed measurements of the intensity distribution of the beam at distances far from the best focus as well as near the best focus and evaluation of XFEL source size and beam quality factor M^2^. Our measurements also support the theoretical prediction that for X-ray photons with energies ~10 keV the radius of the generated photoelectron cloud within the LiF crystal reaches about 600 nm before thermalization. The proposed method has a spatial resolution ~ 0.4–2.0 μm for photons with energies 6–14 keV and potentially could be used in a single shot mode for optimization of different focusing systems developed at XFEL and synchrotron facilities.

Powerful X-ray sources can pump and probe exotic material states with high densities and multiple inner-shell electronic excitations. Unique properties of high-intensity X-ray free-electron lasers (XFELs) to probe matter on the atomic length and femtosecond time scales give new opportunities in such type of investigations. Recently XFEL beams achieved ultra-high (up to 10^20^ W/cm^2^) intensities^1^,^2^,^3^,^4^, which allowed using them succesfully for different new applications: study of the femtosecond electronic response of atoms^5^, demonstration of the possibility of atomic inner-shell X-ray laser generation at 1.46 nanometres^6^, use of XFEL beams for creation and diagnosis of a solid-density plasma^7^. Additionally, the ultra-high intensity of XFELs has extended the frontier of nonlinear optics into the hard X-ray region and sum-frequency generation^8^, parametric down conversion^9^ and third order processes such as two-photon absorption of a 5.6 keV XFEL beam by germanium^10^ have already been reported.

The ability to fully characterize the ultrashort, ultra-intense pulses at XFELs is a crucial task not only for all above mentioned experiments, but also for experiments dedicated to single-molecule or protein nano-crystallography X-ray imaging^11^,^12^,^13^. It requires to provide measurements of the XFEL beam energy distribution after the focusing system with high accuracy, because it is of prime importance both for correct evaluation of the X-ray fluence in the different cross-sections of the beam and for future improvements of the quality of various focusing systems, such as Kirkpatrick-Baez (KB) mirror systems^2^,^4^,^13^,^14^,^15^,^16^, Fresnel zone plates^1^ or a set of parabolic refractive X-ray lenses^3^, which are now typically applied at XFEL or synchrotron facilities for such purposes.

For highly focused X-ray beams the precise characterization of their focusability is a complicated problem. Accurate evaluation of the intensity distribution around the focal point is necessary not only for an accurate determination of the spot size, but also for understanding the quality of the beam and the focusing elements used in particular experiments. The exact beam shape should also be qualified before its application as a nanoprobe or nanopump source. Different methods are used to examine the XFEL beam parameters. For example, a grating interferometer, utilizing the Talbot effect, which enables to obtain wave front profiles of the XFEL beam and to measure the extremely small focal spot size, was developed for characterization of the SACLA beam^17^,^18^. Another sophisticated approach for measurements of the focal spot size is based on the knife-edge scan method^19^,^20^. The recently developed ptychrographic imaging technique^3^,^21^,^22^ allowed to perform the full characterization of a nanofocused XFEL beam, i.e. to obtain the full caustic of the beam, to identify the aberrations of the optics and to measure the wave field for individual pulses.

All above mentioned methods have a very high spatial resolution of the order of 10 nm or better, but have also various drawbacks. For example, some of them require complicated retrieval procedures or could not provide single shot information about the spot size distribution of the focused XFEL beam along the focusing caustic and, in particular, along the XFEL beam propagation direction after entering the target. At the same time such information is crucial for the correct evaluation of parameters in many investigations such as: formation of various phases of matter under extreme conditions; surface nano modification and ablation of solid materials under hard X-ray irradiation; investigations in the field of high energy density science.

We propose here an uncomplicated, in practice easily realized approach for characterization of the focusing caustic of a XFEL beam near the focal spot and inside bulk material–the direct XFEL beam irradiation of LiF crystals or films. It is well known that LiF, as other alkali halide crystals, can host different types of stable color centers (CCs), produced under bombardment by ionizing radiation, like high energy photons and elementary particles, gamma and hard x-rays, neutrons, electrons, and ions^23^,^24^. Under optical excitation by properly selected pumping by UV light, several types of CCs in LiF emit light in the visible spectral range at room temperature. Since the dimension of the single CC is less than 1 nm, and the CCs concentration can reach values of the order of 10^19^–10^20^ cm^−3^, three-dimensional images with high spatial resolution (smaller than 1 micron) can be generated by the ionizing radiation in the LiF material. At the same time, final spatial resolution of the obtained images will usually depend on the spatial resolution of the readout system and could reach 50 nm in the case of using a scanning near field luminescence microscope^25^ or ~200 nm, when confocal luminescence microscopy is applied^24^. LiF crystal or film X-ray detectors were previously successfully used for 2D characterization of the focusing properties of EUV radiation of transient–collisional soft X-ray lasers^26^, of the beams of high-order harmonics generated in plasmas irradiated by relativistic laser beams^27^,^28^ and of a SASE-FEL beam working in VUV mode^29^,^30^,^31^. Recently it was demonstrated^32^,^33^,^34^,^35^ that LiF could be a promising candidate for a hard X-ray detector with high spatially resolution and large dynamic range.

Here, we report, that by means of the direct irradiation of a lithium fluoride (LiF) crystal, *in situ* 3D visualization of the SACLA XFEL focused beam profile along the propagation direction is realized, including propagation inside photoluminescence solid materials. High sensitivity and large dynamic range of the LiF crystal detector with submicron spatial resolution allowed measurements of the intensity distribution of the beam at distances far from the best focus and evaluation of the XFEL source size and beam quality factor M^2^. Our measurements also support the theoretical prediction that for X-ray photons with energies ~10 keV the radius of the generated photoelectron cloud in the LiF crystal reaches about 600 nm before thermalization. The applied experimental method is precise, has a spatial resolution ~0.4–2.0 μm for photons with energies 6–14 keV, not time consuming and realized by using simple and compact hardware and potentially allowed to obtain information in a single shot mode.

## Results

**Figure 1:**
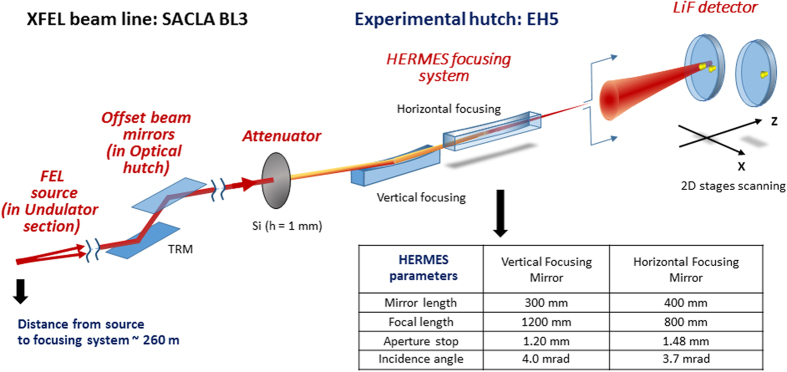
Schematic view of the SACLA-Spring-8 X-ray beam line BL3^39^ and the experimental setup of LiF crystal detector illumination. The SACLA X-ray laser source is generated inside the undulator, corrected by offset beam mirrors, attenuated by 1 mm thickness Si plate and focused by the Hermes KB mirror system^4^ to the surface of the LiF crystal X-ray detector. The LiF crystal had a diameter of 20 mm, thickness of 2 mm and was polished from both sides to optical quality. It was mounted near the focal plane on 2D stages, which allow to move it along the optical axis (axis Z) for changing of the focusing position and perpendicular to it (axis X) for providing a fresh surface of the LiF detector in each new focusing position. Parameters of the HERMES focusing system are presented in the table.

Experiments have been carried out at the hard X-ray beam line BL3 of the SACLA-SPring-8 facility consisting of the undulator section, the electron-beam dump, the optical hutch and the experimental hall EH5. The schematic view of the experimental setup is shown in Fig. 1. In our experiments the XFEL operated at the following conditions: photon energy is E_ph_ = 10.1 keV, pulse energy at the exit of the undulator is E_XFEL_ = 400 μJ and repetition rate is 30 Hz. A Si plate attenuator decreased the energy of the pulses by a factor of 1500. The XFEL beam was focused by a Kirkpatrick-Baez (KB) type HERMES focusing system, which was specially designed for operation at the EH5 optical line. The transmission of energy due to the reflections of the beam on the mirrors of the focusing system amounted to 34 percent. Thus the energy of XFEL beam pulse delivered to the LiF crystal surface, which has been used as X-ray detector, was about 90 nJ. Independent measurements of the focal spot of the KB HERMES focusing system was done using a wire scan technique and demonstrated that the focal spot is of ~0.06 μm^2^. It means that the flux density on the surface of the detector did not exceed 1.5 μJ/μm^2^. Such value is ~3 times less than the damage threshold for dielectrics measured in ref. 36. An additional check by a differential optical microscope confirmed the absence of any damage on the surface of the LiF crystal even after its irradiation at the best focus position.

**Figure 2:**
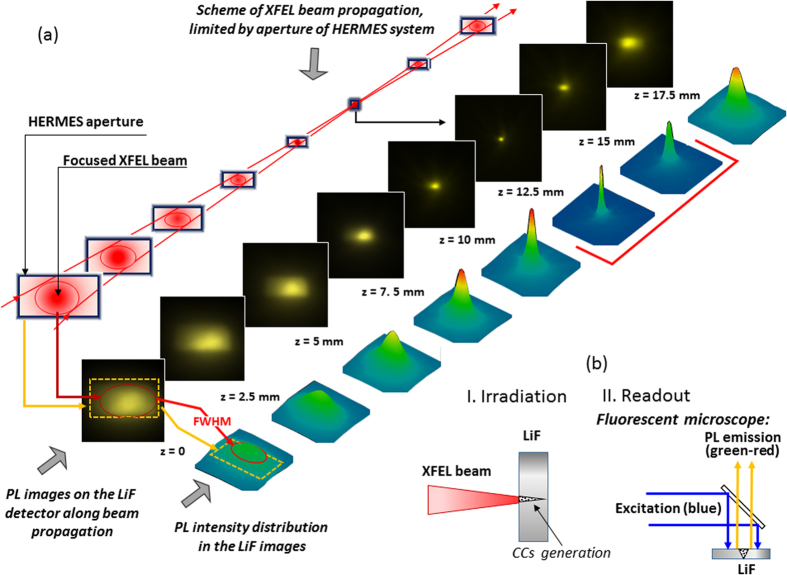
Measurements of intensity distribution of the XFEL beam near the focus position. **(a**) Central diagonal-sequence of PL images, obtained in 10 XFEL shots on the surface of LiF detector in different planes; upper diagonal-schematic interpretation of the PL images; below diagonal-3D plots of the PL intensity for corresponding images. To avoid saturation, two images near the focus, marked by the red line, were readout with 5 times less accumulation time compared to all others. **(b)** Schematical principals of the LiF crystal detector application for hard X-ray imaging. In the first step (I) the X-ray radiation of the XFEL beam irradiated the LiF crystal and produced color centers (CCs)^24^, which formed a hidden image according to the intensity distribution of the XFEL beam. In the second step (II) the stored image is visualized by means of CCs photoluminescence. The confocal fluorescent microscope Nikon C2+ was used for the readout process. Objective with magnification of 40^×^ was used, which allowed to digitize images with spatial resolution up to 0.3 μm.

The intensity distribution of the focused XFEL beam was measured in air at sequences of planes near the focal point (See Fig. 2(a)). For that purpose the LiF crystal was moved along the axis Z over a range of 17.5 mm with a stepsize of 2.5 mm. For each step, the LiF was also shifted in the X-direction to irradiate the crystal on a fresh surface. Ten XFEL shots were used for irradiation of the LiF crystal at each focusing position. We selected such number of shots to be in agreement with the number of shots used in wire scan measurements of KB focusing spot. This allowed us directly to compare the spot size measured by two different methods. It is also necessary to mention that point stability of SACLA XFEL is of the order of ~0.3 μrad. It means that for our focusing system the displacement of the position and size of focal spot was not more than 300 nm due to such factor. Under hard X-ray irradiation specific types of F_2_ and F_3_^+^ CCs are generated in the LiF. The number of generated CCs corresponds to the intensity of incident photons^32^,^33^. CCs are very stable at room temperature, so the LiF stores a hidden image of the XFEL beam intensity distribution in a form of 3D distribution of CCs density for a long time. According to ref. 37 the attenuation length of photons with energy of 10.1 keV in LiF is 675 microns. It means that CCs will be generated not only in a thin layer of the front surface, as it occurs in the case of EUV and soft X-ray irradiation, but also deeply inside the crystal as the XFEL beam propagates through it. So, the XFEL beam passing through the LiF crystals generated CCs with some distribution. The hidden image of such CCs distribution represents a snap shot of the beam intensity distribution along the XFEL beam propagation direction inside the matter (See Sketch I. in Fig. 2(b)). The optical read-out of the stored images by means of LiF crystal photoluminescence (PL) is provided by a high resolution microscope operated in luminescent mode (See sketch II. in Fig. 2(b)).

As it could be seen from the images presented in Fig. 2(a), the beam is focusing, passing through the focus and then diverging. The aperture of the focusing system and the position of the beam axis relative to this aperture also could be seen in the images. A schematic interpretation of PL images and 3D plots of PL intensity is presented above and below of the corresponding images. We would like to stress that high sensitivity and large dynamic range of the LiF detector make it possible to record without any additional attenuation both the intensity profile of the entire beam far from focus (including the very low signal from the boundary of the beam, limited by the aperture of the mirrors), and the intensity profile in the best focus, where the signal increases by a few orders of magnitude. The difference in intensity of the beam in the range of observation was so large, that we are faced with the problem of limited dynamic range of the microscope readout system. To avoid saturation of the PL signal in the images obtained near the focus (two images marked by red line in Fig. 2(a)), it was necessary to reduce the accumulation time T_accum_ for them to almost one quarter compared to the accumulation time for other images.

**Figure 3:**
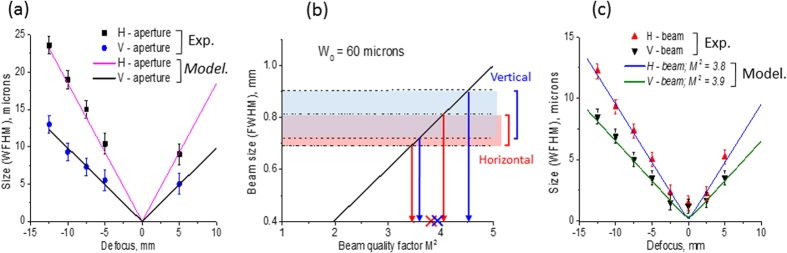
Size of the HERMES focusing system apertures projection and XFEL beam vs defocusing (experiment and calculations). Panel (**a**): comparison of vertical and horizontal sizes of the HERMES aperture measured along the beam propagation with calculated ones using geometrical optics. Panel (**b**): Calculated dimension of the Gaussian beam incident on the HERMES mirrors *vs* the beam quality factor M^2^ for the source size W_0_ = 60 μm. Shaded areas show, estimated according to the experimental data, the values of the beam quality factor M^2^ of the XFEL beam incident on the HERMES mirrors in horizontal (pink) and vertical (blue) directions. Arrows show the corresponding ranges of the beam quality factors for the case of W_0_ = 60 microns in both directions, which are M^2^_H_ ~3.5 ÷ 4.0 and M^2^_V_ ~3.6 ÷ 4.5. Panel (**c**): Experimental and calculated dependences of beam size W’_exp_ *vs* the distance Δf from the focal plane of the HERMES system, independently for horizontal and vertical directions. Good overlap of experimental and calculated sizes for M^2^ = 3.8 in horizontal and M^2^ = 3.9 in vertical directions can be seen.

In the sequence of images presented in Fig. 2(a) we can distinguish two features: bright spots, which we suppose are belonging to the XFEL beam itself and less bright, almost homogeneously illuminated rectangular areas around the XFEL beam, which represent the projection of the HERMES mirror apertures in different planes. To ensure that the rectangular contours are the images of the focusing system aperture, we compared the sizes of that contours in the sequence of measured images with the calculated sizes of the beam profile limited by the aperture and propagated to the focal position. In assumption, that the solid angle subtended by the aperture has a vertex in the position of beam waist, the size of the beam in the arbitrary plane between the mirrors and the focus is A’_v; h_ = A _v;h_ δf /F_v;h_, where A is the size of the aperture stop, δf is the distance from the focal plane, F is the mirror to focal distance, and subscripts v; h are related to vertical and horizontal directions, respectively. We calculated A’_v_ and A’_h_ and compared them with experimental data measured from the PL images. As can be seen in Fig. 3(a), the results are in a very good agreement. The obtained result confirms the fact that the observed rectangular frame is the image of the HERMES mirror apertures and allows us to determine the ratios between the sizes of the aperture projections and the apparent sizes of the beam along the beam propagation as 0.7 ± 0.07 for vertical and 0.5 ± 0.05 for horizontal directions. Using the obtained results we measured vertical and horizontal sizes of the focusing system aperture and the XFEL beam in dependence of z-position of the detector (Fig. 3(a,c)).

In accordance with the theory of Gaussian laser beam propagation, it is sufficient to know the size of the waist or the divergence of the beam and the wavelength to determine all the beam properties. For a non-ideal Gaussian beam the parameter M^2^ of laser beam quality, which specifies the degree of variation of the actual beam from the ideal one, needs also to be employed. But, so far the XFEL source parameters, such as source size and M^2^, have not yet been completely investigated and their measurements remain important. Following the theory of Gaussian beams^38^ the size W_Z_ of the input beam (to FWHM of the intensity on axis) at distance Z from its waist is


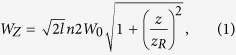


where W_0_ is the beam radius at the waist (Z = 0) and Z_R_ = πW^2^_o_/λM^2^ is the Rayleigh range, M^2^ is the beam quality factor. In accordance with the results of wavefront measurements^18^ the SACLA XFEL beam propagated from the source point located inside of the undulator at a distance of ~55 m before the end of the last section. Our focusing system was placed at a distance of 205 m downstream of the undulator. So, we can assume that in our experiment the distance from the source point to the focusing system is s ~260 m. Another important parameter for modeling the beam propagation is the waist radius and beam quality factor M^2^.

Using expression (1) we calculated the size of the input XFEL beam on the mirrors of the focusing system in dependence of the factor M^2^ for our above mentioned experimental conditions, assuming the size of the beam waist W_0_ = 60 μm according with previous measurements^2^. The correspondent plot (black line) is shown in Fig. 3(b). For this plot we imposed rectangular areas, corresponding to the size of the beam in vertical (blue) and horizontal (pink) directions, obtained by the procedure described above, based on the experimental measurements. The cross section of the plot with these areas defined the range of the beam quality factor M^2^ for selected beam waist W_0_. From Fig. 3(b), we could estimate that, for our experimental conditions the beam factor M^2^ was in the following ranges : M^2^_H_ ~3.5–4.0 for horizontal direction and M^2^_V_ ~3.6–4.5 for the vertical direction, to satisfy such the beam waist W_0_.

For a more precise determination of the XFEL beam quality the comparison of the calculated beam caustic near the focal plane with the experimental one has been provided. It is seen from the images in Fig. 2(a) that despite of a small decentering of the beam, the aperture of the mirrors practically does not limit the aperture of the beam. In such case diffraction effects can be neglected. According to ref. 38, in assumption of geometrical optics for the diffraction limited focusing system, the Gaussian profile of the output beam could be determined as


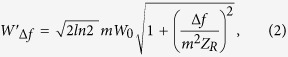


where W′_Δf_ is the size of the focused beam (FWHM of the beam intensity on axis), Δf is the distance from focal plane, and 
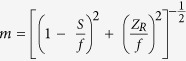
 is the transvers magnification.

Applying equation (2) for different values of the beam quality factor in the range M^2^ = 3.5–4.5, we calculated the path of the beam from the source with determined above the waist radius W_0_ = 60 μm (i.e. 30 μm in *rms*) through the HERMES focusing system in horizontal and vertical directions. We found that the best fit of the experimental results takes place in the case of M^2^ = 3.8 (blue solid line) for horizontal and M^2^ = 3.9 (green solid line) for vertical directions. The comparison of the calculated beam caustic near the focal plane with the experimental one is shown in Fig. 3(c) and shows good agreement. We should mention that values of the beam quality factor, measured here are slightly larger than it was distinguished in previos experiments^2^. This may be connected with the different conditions of the accelerator adjustment^39^,^40^ in our experimental run.

**Figure 4:**
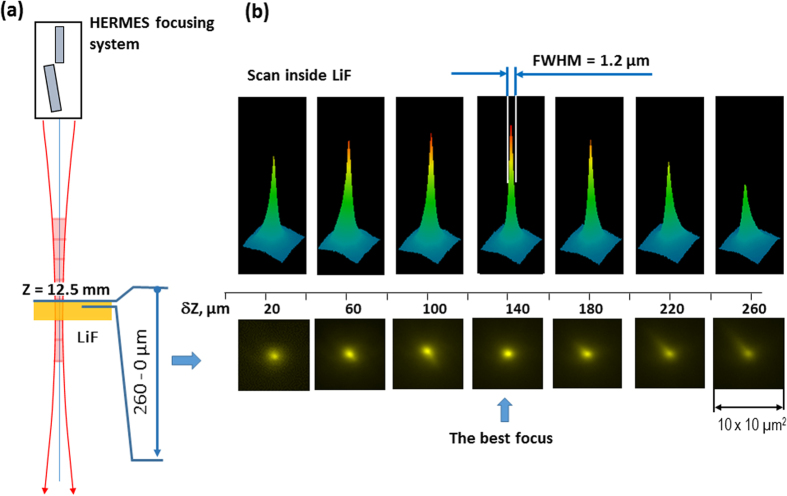
Observation of the focused XFEL beam propagation through the waist inside the LiF crystal. **(a**) Sketch of the measurements showing the range of depth where measurements have been done. Step of scan δZ was of 40 μm. The XFEL beam with photon energy of E = 10.1 keV was focused by KB mirrors on the LiF crystal at the best focusing position of Z = 12.5 mm, which were determined by a long distance preliminary scan (See Fig. 2a). (**b**) PL images, measured at different distances from the surface of the LiF crystal with magnification of 40^×^ (bottom row) and the corresponding intensity distribution plots (upper row). Presented images give information about the 3D intensity distribution of the XFEL beam propagating inside the LiF crystal.

As we could see from the images in Fig. 2(a) the best focusing position in our experiments was around Z = 12.5 mm. We would like to remind that each image actually is a 3D image, because it keeps information not only about the XFEL beam intensity distribution on the surface of the crystal, but also along the XFEL beam propagation inside it. In such cases by providing scanning of the PL images inside the LiF crystal we could find the position of best focusing of the XFEL beam with higher accuracy. Such scanning was carried out with the same readout parameters and magnification of the microscope of 40^×^ at different (up to 260 μm) depths inside LiF crystal. The obtained images are presented in Fig. 4(b). As could be seen from the images the peak intensity of PL increases with the depth of the XFEL beam propagation (despite the fact that the XFEL beam intensity absorption is also increasing) and reaches a maximum at the distance δZ = 140 μm from the surface. A maximum of the PL intensity in the image downstream of the convergent XFEL beam defines the best focal plane of the focusing system. So, applying a two-stage readout procedure of the LiF crystal luminiscence we have found the position of the best focal spot Z_F_ = 12.5 mm (first stage- PL readout on the surface of the crystal) + 0.14 mm (second stage- PL readout inside the crystal) = 12.64 mm with high accuracy of +/−40 microns relatively to the initial position of the detector at z = 0.

**Figure 5:**
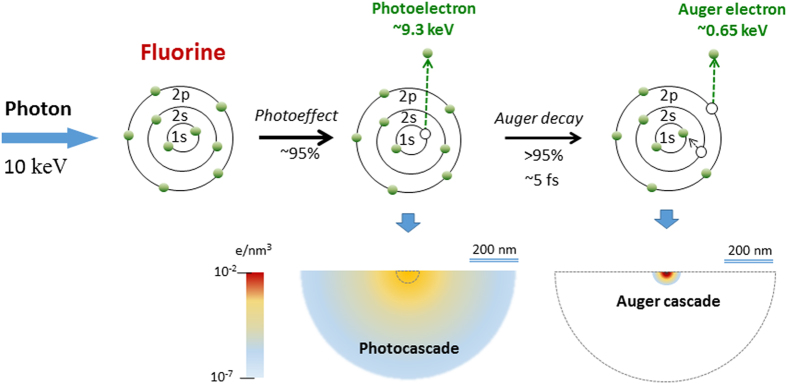
Sketch of the electron cascades in LiF caused by interaction of XFEL photons with fluorine ions. The limit of resolution defined by the resolution of the microscope system (~0.9 μm in our case) and the secondary processes (mainly photoionization) caused by interaction of 10 keV photons with LiF according to modeling provided in ref. 42, the size of the secondary electrons cloud should be within a diameter of ~1.2 μm, which is in a good agreement with the experimentally measured value of smallest focusing spot.

We should stress that, according to Fig. 4(b) the smallest size of the focal spot, measured in the best focus position, is ~1.2 μm. This is a greater value, than the one measured in our experiments using the wire-scan method, which gives a value of the focal spot ~200 nm × 300 nm. As both measurements were done using the same number of shots it is obvious that the focal spot measured by the LiF crystal is essentially larger than the actual one. A natural question which arises is why such a large discrepancy between the two different methods of measurements appeared? One of the possible reasons could be the limit of the spatial resolution of microscope used for the read-out of the PL images. But in our case the spatial resolution of read-out process was essentially lower of ~0.3 μm. Another possible reason why the diameter of the XFEL beam focal spot measured by PL luminescence is larger than the actual XFEL beam focus spot is the influence of the secondary electron cascade^41^ generated in the LiF crystal by the incoming X-ray photons. Indeed, the dominating process in the LiF after the 10.1 keV X-ray photons enter the crystal is photoionization of the K-shell of fluorine producing ‘fast’ photoelectron with energies of about 9.4 keV (See Fig. 5). The formed K-hole is filled then by the Auger decay producing ‘slow’ Auger electrons with energies of about 650 eV. The fast photoelectrons and the slow Auger electrons further interact with the crystal producing other electrons, developing a cascade, and exciting the LiF, which gives the luminescence. Detailed simulation of the cascade is a laborious calculation task and is beyond the scope of the present manuscript. Recently a similar cascade has been calculated in^42^ for the urea crystal for 8 keV photoelectrons and 0.4 keV Auger electrons. The radius of the electron cloud generated by the photoelectrons before thermalization reaches 600 nm, while for the Auger electrons it is approximately 20 nm. Having in mind the similarity in the photoelectron energies and the fact that fluorine (Z = 9) is a neighbor of C, N, O (Z = 6–8), which are the main constituents of the urea crystal, in the periodic table, one should expect qualitatively similar dimensions of the electron clouds in the urea and the LiF crystals. Indeed, the radius of the cloud predicted for the urea crystal is in excellent accordance with our observations in LiF.

**Figure 6:**
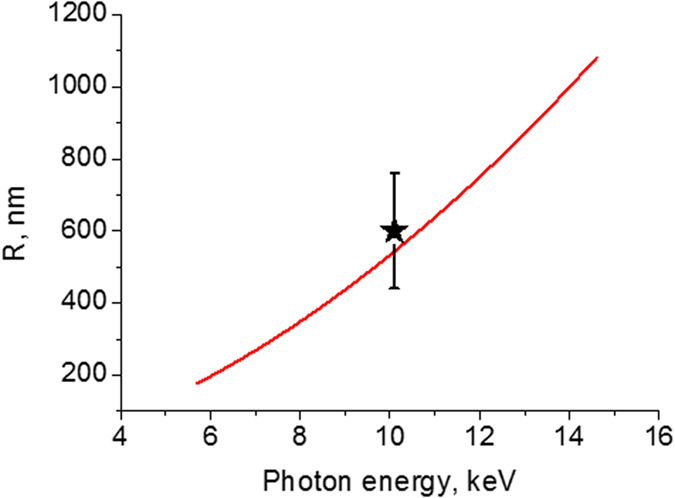
Average penetration depth R of the fluorine K-electrons in LiF crystal as function of the incident photon energy. The black star indicates the experimental point, evaluated from the limit of spatial resolution in the fluorescent images of XFEL beam.

Since it seems logical that the radius of the spot roughly corresponds to the radius of the electron cloud, one can try to estimate it by semi-empirical formulas used for treating the passage of electrons through matter^43^. As a result of multiple scattering, the trajectory of the electrons in LiF is rather wiggly than straight. Universal parametric formulas have been established, on the basis of Monte Carlo simulations of electron transport, supported by experimental data, for ‘average penetration depth’, ‘extrapolated range, ‘average reach’ and other related quantities for the electron energies higher than 100 keV (for example^44^,^45^,^46^). Electrons with the energies lower than 100 keV have a range of the order of only a few micrometers in condensed materials and there is often little interest for the applications in such a small energies. Nevertheless in some cases the above formulas have been used for the lower electron energies^43^,^44^,^47^. It is also not clear which of the possible quantitative measures of the distributed electron cloud is the most useful in our case. After the photoabsorption, the fluorine K-shell photoelectron is ejected preferentially perpendicular to the photon beam, surrounded by the sample. We found that the average penetration depth, *z*_*0*_, which is the average of the endpoint depths of the fluorine K-electrons, describes well the radius of the observed spot. To calculate the factor d = *z*_*0*_/ *r*_*0*_, where *r*_*0*_ is the continuous slowing-down approximation mass range (the average path length traveled by an electron until it comes to a stop), by an interaction database EMID^48^, created by T. Tabata and coworkers, we use the effective nuclear charge Z_*eff*_ = 7.4998 and the effective mass number A_*eff*_ = 16.212 for LiF, as obtained by standard prescriptions (e.g.^44^,^46^). Thus obtained *d* was multiplied by the value of *r*_*0*_ found according to parametric expressions from^44^ with the mean excitation energy 94 eV of LiF^49^ and, finally, multiplied by the LiF density of 2.64 g/cm^−3^. The resulting absolute average penetration depth R of the fluorine K-electrons as function of the photon energy is shown in Fig. 6. The predicted radius of the spot increases with increasing the energy of the photon. For the photon energy in our experiment (10.1 keV) the calculations give R = 540 nm in a good agreement with our measurements. We performed the similar analysis and calculations of R for the urea crystal and obtained R = 700 nm for the electron energy of 8 keV, which is in satisfactory agreement with *ab initio* treatment in^42^. It is expected that our predictions should be more reliable for the higher photon energies. It would be interesting to check the predicted tendencies of the spot diameter in future measurements of the luminescence in LiF for other photon energies.

## Discussion

In this work we show that a LiF crystal, which is now well known as a very efficient detector for the soft X-ray spectral range, can be effectively used for *in situ* (the detector is placed in the direct beam, where focusing is occurred) 3D recording of the focused XFEL beam structure around the focal plane in a simple, very compact scheme with a spatial resolution ~0.4–2.0 μm for photons with energies 6–14 keV. Due to the high spatial resolution, the very large dynamic range and the long penetration depth of the XFEL radiation inside the LIF crystal, the experimental results allowed to measure *in situ* a caustic of the beam, and to provide a high precision determination of the focal plane with an accuracy of +/−40 microns. We should stress a drawback of our measurements. The detector after exposure needs to be removed from the beamline to be looked under a high-quality fluorescence microscope for 3D visualization. It means that presented method is not *on line* and this leads to a not so rapid diagnostic in the end and is relatively time-consuming.

Moreover, using the LiF crystal enables detailed measurements of the influence of the background radiation. High sensitivity and large dynamic range of LiF crystal X-ray detectors allow successfully using it for measurements of the XFEL beam intensity profile not only in the focal plane inside the target, but also for the characterization of unfocused XFEL beam in a single shot. Such possibility was confirmed in our recent experiments, when high quality images were obtained in a single shot mode even in the case of XFEL beam intensity on the detector as low as ~1 nJ/μm^2^. Additionally such technique gives the possibility to record any diffraction patterns if they will appear in the propagated beam due to the touching of edges or any obstacles by XFEL beam. To conclude, the unique behavior of the LiF crystal to response for deposited X-ray doses by proportional generation of color centers density inside the crystal volume, its high resolution, high dynamic range, high radiation damage threshold, absence of any electronic circuits, insensitivity to visible light and relatively low cost give enormous advantages in using this new diagnostic technique for visualization and optimization of both coherent and non-coherent hard X-ray sources, compare with CCDs detectors, photographic films, and photoresists.

## Additional Information

**How to cite this article**: Pikuz, T. *et al.* 3D visualization of XFEL beam focusing properties using LiF crystal X-ray detector. *Sci. Rep.* **5**, 17713; doi: 10.1038/srep17713 (2015).
